# Systematic review of the tools of oral and dental health literacy: assessment of conceptual dimensions and psychometric properties

**DOI:** 10.1186/s12903-020-01170-y

**Published:** 2020-07-03

**Authors:** Mohtasham Ghaffari, Sakineh Rakhshanderou, Ali Ramezankhani, Yadollah Mehrabi, Ali Safari-Moradabadi

**Affiliations:** 1 Department of Public Health, School of Public Health and Safety, Shahid Beheshti University of Medical Sciences, Tehran, Iran; 2 Department of Epidemiology, School of Public Health and Safety, Shahid Beheshti University of Medical Sciences, Tehran, Iran

**Keywords:** COSMIN, Psychometric, Oral health literacy, Dental health literacy

## Abstract

**Background:**

This article aims to provide a description of conceptual dimensions and psychometric properties of the tools of oral and dental health literacy.

**Methods:**

Two authors in this study conducted electronic searches in the Medline (via PubMed), and Embase databases to find relevant articles from 1990 to present day. Evaluation of the tools was carried out in two parts; general evaluation of the tools using skills introduced by Sørensen et al., and qualitative assessment of psychometric properties using COSMIN checklist.

**Results:**

After reviewing 1839 articles on oral and dental health literacy and evaluating 33 full text articles for eligibility, 21 articles entered the study. The sample size varied from 20 to 1405 subjects and the items of each tool ranged from 11 to 99 items. Of the 21 tools examined, 16 tools were evaluated for word recognition. For the studies examined, the evaluation of COSMIN scores was often fair or good. Of the 21 tools examined, 9 tools at least in one dimension were in the category of “poor”, 19 tools were in the category of “fair”, 20 tools were in the category of “good”, and 4 tools were in the category of “excellent” in at least one dimension.

**Conclusion:**

The findings of this study showed that some aspects of oral and dental health literacy are being ignored in the existing tools. Therefore, the authors of present study emphasize on the necessity to design and develop a comprehensive tool and take into account two characteristics of simplicity and briefness for international use.

## Background

In the twenty-first century, health literacy (HL) has been introduced as a global issue and a priority in health [1, 2], and the World Health Organization has identified HL as one of the greatest determinants of health [3, 4]. One of the important topics in the field of health, is oral and dental health. Oral and dental health literacy is a subset of HL [5]. Using health literacy, the most common definition of OHL is “a degree of people’s ability to obtain, process, and understand oral health information and make appropriate oral health decisions” [6]. Oral and dental health literacy skills are important for reducing oral health inequalities and promoting oral health information [7].

Some studies point to the link between low level of OHL and lack of using preventive or therapeutic services and also understanding of health information transferred by the health care providers [8, 9]. The American Dental Association has confirmed that limited HL is an obstacle to the prevention, diagnosis and treatment of oral and dental illness, and clear, accurate and effective communication is one of the essential skills for effective dental practice [10]. There is strong evidence about the economic costs associated with the low level of oral and dental health literacy [11, 12], and various studies have referred to the convergence between oral health and general health and the effects of poor oral health on quality of life [11, 13, 14]. So, there are many challenges in educating and helping people to obtain the necessary resources to make decision about oral and dental health. Clear communication in plain language about oral health and services will help to improve oral health [15]. On the other hand, level of knowledge about the importance of HL in oral and dental health has increased dramatically in recent years, and efforts have been made to integrate the concept of HL in oral health research [16–18].

### Measuring oral and dental health literacy

Dickson-Swift et al. (2014) explained the primary tool for OHL has been derived from the HL tools. For example, the tool of Rapid Estimate of Adult Literacy in Dentistry (REALD) is an adaptation of the Rapid Estimate of Adult Literacy in Medicine (REALM), [19]. Similar examples include the Test of Functional Health Literacy in Dentistry (ToFHLiD), which has been adopted from the Test of Functional Health Literacy in Adults (ToFHLA), [20]. Primary tools received similar criticisms about the general health literacy versions because they were first the word recognition tools that did not actually measure oral and dental health literacy, but rather they measured the reading skills of oral health contents [21]. A wide range of similar tools has been designed to display, diagnose and measure OHL. However, there is currently no tool available as a gold standard for oral and dental health literacy [22]. Due to the predicted increase in the number of adults in the world and the low level of oral and dental health literacy in this population, as well as the correlation between OHL and the probability of taking preventive interventions, it is vitally important to prioritize the accurate assessment of oral and dental health literacy.

So far, only one systematic review has been carried out to evaluate the oral and dental health literacy tools in 2013 [23], which examined the HL tools in general. Therefore, the present study intends to review and examine the HL tools in terms of dimensions and psychometric evaluation using the COSMIN checklist, by updating the study of Sørensen et al. (1990 to present). We expect the findings of this study to be effective in identifying and selecting the most appropriate tool for various purposes.

## Methods

We report this manuscript in accordance with the Preferred Reporting Items for Systematic Reviews and Meta-analysis (PRISMA statement) guideline [24] (Supplementary material 1).

### Search strategy

A systematic library search was conducted by one of the authors (ASM) in consultation with a librarian across seven electronic databases (CINAHL, Embase, PsycINFO, PubMed, Scopus, Web of Sciences and LIVIVO). Gray literature sources was searched in the System for Information on Grey Literature in Europe (SIGLE) (http://www.opengrey.eu/). A hand-search of relevant bibliographies was performed to identify potential studies that were excluded. The key words used in the search included; oral, dental, Health, Literacy, tool, instrument, questionnaire, Psychometric, validity, reliability. Only peer-reviewed articles that were written in English were considered. The full search strategy for each database is provided in Table 1.

**Table 1:** Library Search Strategy

Database	Search terms	Results
CINAHL	(TI (Oral* OR dental*) OR AB (Oral* OR dental*)) AND (TI (Health AND Literacy) OR AB (Health AND Literacy)) AND (TI (tool OR instrument OR questionnaire) OR AB (tool OR instrument OR questionnaire)) AND (TI (Psychometric OR validity OR reliability) OR AB (Psychometric OR validity OR reliability))	349
EMBASE	(‘Oral*’:ab,ti OR ‘dental*’:ab,ti OR ‘Oral ‘/exp. OR ‘dental’/exp) AND ((‘health’:ab,ti AND ‘literacy’:ab,ti OR (‘health ‘/exp. AND ‘literacy’/exp)) AND (‘tool ‘:ab,ti OR ‘instrument’:ab,ti OR ‘questionnaire’:ab,ti OR ‘tool’/exp. OR ‘instrument’/exp. OR ‘questionnaire’/exp) AND (‘Psychometric’:ab,ti OR ‘validity’:ab,ti OR ‘reliability’:ab,ti OR ‘Psychometric’/exp. OR ‘validity’/exp. OR ‘reliability’/exp)	458
PSYCINFO	(TI (Oral* OR dental*) OR AB (Oral* OR dental*)) AND (TI (health AND literacy) OR AB (health AND literacy)) AND (TI (tool OR instrument OR questionnaire) OR AB (tool OR instrument OR questionnaire)) AND (TI (Psychometric OR validity OR reliability) OR AB (Psychometric OR validity OR reliability))	214
PUBMED	(((((Oral [MeSH Terms]) OR dental [MeSH Terms])) AND ((Health [MeSH Terms]) AND Literacy [MeSH Terms])) AND (((tool [MeSH Terms]) OR instrument [MeSH Terms]) OR questionnaire [MeSH Terms])) AND (((Psychometric [MeSH Terms]) OR validity [MeSH Terms]) OR reliability [MeSH Terms]) OR (((((((Oral [Title]) OR dental [Title]) OR Oral [Abstract]) OR dental [Abstract])) AND ((((Health [Title]) AND Literacy [Title]) OR Health [Abstract]) AND Literacy [Abstract])) AND ((((((tool [Title]) OR instrument [Title]) OR questionnaire [Title]) OR tool [Abstract]) OR instrument [Abstract]) OR questionnaire [Abstract])) AND ((((((Psychometric [Title]) OR validity [Title]) OR reliability [Title]) OR Psychometric [Abstract]) OR validity [Abstract]) OR reliability [Abstract])	495
SCOPUS	(TITLE-ABS-KEY (oral* OR dental*)) AND (TITLE-ABS-KEY (“health” AND literacy”)) AND (TITLE-ABS-KEY (tool OR instrument OR questionnaire)) AND (TITLE-ABS-KEY (Psychometric OR validity OR reliability))	341
LIVIVO	(TI = (((MESH=Oral OR MESH = dent*) AND MESH=Health AND MESH = Literacy) AND (MESH = tool OR MESH = instrument) OR MESH = questionnaire)) OR (((TI = (Oral* OR dent*)) AND TI = (Health AND Literacy)) AND TI = (tool OR instrument OR questionnaire)) AND (Psychometric OR validity OR reliability)	453
WEB OF SCIENCE	(((TI = (Oral* OR dent*) AND TI = (Health AND Literacy) AND (tool OR instrument OR questionnaire))) OR ((((TS = (Oral* OR dent*) AND TS = (Health AND Literacy) AND (tool OR instrument OR questionnaire) AND TS = (Psychometric OR validity OR reliability))))	270
Open Gary	(((oral* OR dental*) AND ((health AND literacy) AND (tool OR instrument OR questionnaire) AND (tool OR instrument OR questionnaire)))	0
Bibliography		15
	Total	2595

Studies that fulfilled the following criteria were included: (1) Studies whose results have assessed one or more of the following psychometric properties: internal consistency, reliability, measurement error, content validity, face validity, structural validity, hypothesis testing, cross-cultural validity, criterion validity, or responsiveness; (2) all studies published between each database’s inception and January 2019 that have been design, develop, or psychometric to measure oral and dental health literacy; and (3) studies published in English language. Studies were excluded if they were (1) conference abstracts, systematic review and meta-analysis, and other studies that did not meet the inclusion criteria as well (2) Protocol studies related to psychometrics (Studies with no results).

### Screening, data extraction

Search strategies were performed by two trained authors (ASM and SR). The authors were the same at all stages of the study. In the first stage, titles and abstract of the articles were evaluated. In the second stage, the full text of the articles was independently reviewed by two authors. To assess agreement between reviewers for study selection, we used the Kappa (K) statistic, which measures agreement beyond chance [25]. A Kappa value > 0.6 is considered substantial agreement and a Kappa value > 0.8 is considered almost perfect agreement [26]. The quality of each article was quantified by a score of 0 or 1 (low or high).

### Assessment of risk of bias

The COSMIN Risk of Bias checklist was used to assessment the methodological quality of the included studies on measurement properties. This checklist consists of several boxes, each pertaining to a specific measurement property and containing several questions/standards about the design requirements and statistical methods of the studies. For each measurement property in each study, the COSMIN item with the lowest score will indicate the overall methodological quality (i.e., worst-score-counts method) [27, 28].

In this study, the End Note software was used to organize the references. Data extraction included author, year, target population, sample size, location of the study, complete instrument name, report, time management (min), number of questions and scales, and rating. One part of the data extraction is related to the process of qualitative evaluation of the tools which is discussed below. The searches conducted From February to April 2019. The authors entered the data existed in the articles into Excel software based on the items in the data extraction section.

### The process of qualitative evaluation of the studies related to OHL tools

At this stage, the full texts selected related to OHL tools at the screening stage were evaluated by two authors (ASM and MGH) independently, and on the basis of two factors. Differences in judgment were resolved through a consensus procedure.

1. Evaluate aspects of OHL: To examine the specific skills and competencies measured by the different tools we used the taxonomy of skills identified by Sørensen et al., 2012 in their content analysis of health literacy definitions. This process evaluates the tools based on different dimensions, including the reading dimension (basic skills for reading based on the International Student Assessment [PISA]), interactive dimension (the ability to communicate about health issues), perceptual dimension (the ability to extract meaning from information sources), and computational dimension (the ability to perform numeric tasks and mathematic operations). The remaining dimensions includes; information search (which requires the ability to find information on health for health management), performance (the ability to use and process, or act upon health information and informed decision), assessment (ability to filter, change and evaluate information), and responsibility (the ability to take responsibility and make decision on health and Health care), [23].
2. Qualitative assessment of methodology and psychometric properties. To evaluate the psychometric section, the COSMIN checklist (the consensus-based standards for the selection of health measurement instruments) was used [29]. This tool examines the quality of studies in 4 areas, 12 domains and 114 items. The 12 domains include; internal consistency, reliability, measurement error, content validity, structural validity, hypothesis testing, cross-cultural validity, criterion validity, responsiveness of theory methods (if applied), interpretability, and generalizability of the tool’s properties. Since there is no gold standard for the oral and dental health literacy tools [22], the domain of Criterion validity was not considered. All 114 items were evaluated according to the poor, fair, good, and excellent scale. Taking the lowest rating for each item in one box, an overall quality score (poor, fair, good, excellent) is obtained for each measurement property separately [28, 30].

Supplementary material 2 presents the ratings of the quality of each instrument based on the COSMIN checklist as well as three categories of ‘adequate’, ‘not adequate’, and ‘unclear’ [29].

### The strength of evidence assessment

To evaluate the instruments, the strength of evidence for each was rated on a scale graded as strong, moderate, limited, conflicting or unknown. The criteria for rating were the methodological and measurement quality, the number and consistency of results among the body of research using the instrument. Strong evidence was marked by several articles with high quality methods or one published paper of an excellent quality and a report of consistency of the properties. Moderate level was characterized by several articles with fair methods or one published paper of good quality. Limited rating would characterize an instrument with one article of fair quality. Conflicting level would describe an instrument that had mixed findings. Unknown rating was for an instrument with several papers of low quality methods or simply no published paper.

## Results

**Fig. 1:**
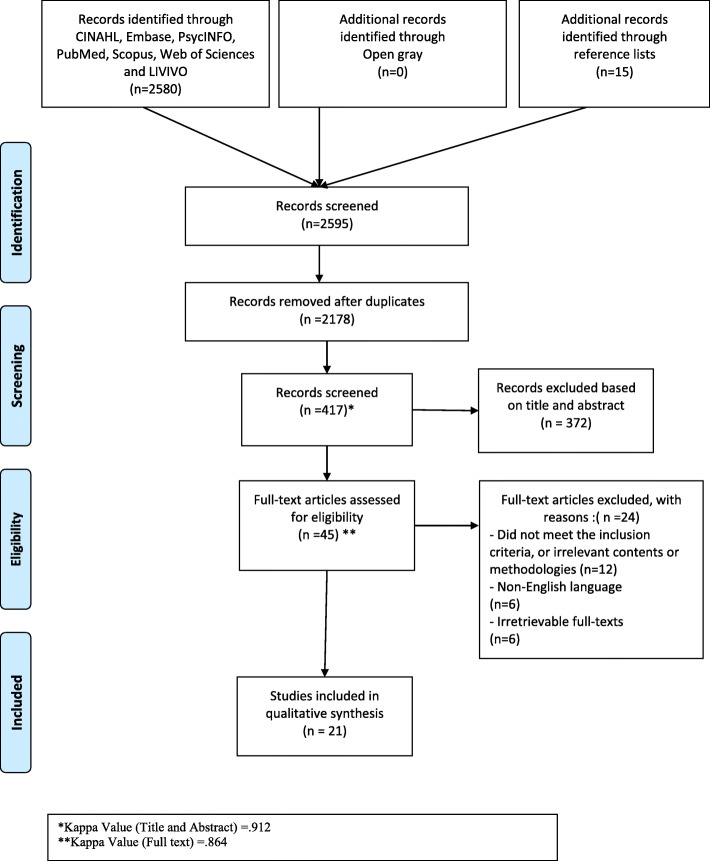
Flowchart of article selection

Two authors screened 291 articles, and the full text of 33 articles. Finally, 21 articles had the criteria to enter the study (Fig. 1).

The sample size varied from 20 to 1405 subjects and items per instrument ranged from 11 to 99. Most studies had examined the adult age group. A detailed description of the measurement tools is shown in Table 2.

**Table 2:** Describing Details of OHL Tools

Author	Instrument	Year Publication	Setting	Characteristics			Psychometrics			Measure style	Assessment	Scoring categories
				Domains assessed	Validation sample population age	Modes of administration in validation study	Number of items	Sample size in validation study	Language of validated version			
Richman et al., [31]	REALD-99	2007	North Carolina	Pronunciation	Adults: 18 to 64 years	Face-to-face	99	102	English	REALM Family	Objective	Sum score (0–99)
Gong et al., [32]	ToFHLiD	2007	North Carolina	Comprehension, Numeracy	Adults: 18 to 64 years	Face-to-face	80	102	English	TOFHLA Family	Objective	Weighted score (0–100)
Lee et al., [33]	REALD-30	2007	North Carolina	Pronunciation	Adults: 18 to 64 years	Face-to-face	30	202	English	REALM Family	Objective	Sum score (0–30)
Sabbahi et al., [34]	OHLI	2009	Toronto	Comprehension, Numeracy	Adults: 18 to 64 years	Paper and pencil, Face-to-face	57	100	English	TOFHLA Family	Objective	Possible range: 0–100 (comprehension score × 1.31, numeracy score × 2.63), with 0–59: inadequate HL, 60–74: marginal HL, and 75–100: adequate HL
Macek et al., [35]	CMOHK	2010	Baltimore	Pronunciation	Adults:aged 45–64 years	Face-to-face	28	100	–	REALM Family	Objective	CMOHK scores were divided into three categories. Scores from 0 to 11 represented“poor,“12–14 corresponded to“fair,” and 15–23 represented “good”
Atchison et al., [36]	REALM-D	2010	USA	Comprehension, Pronunciation	Adults: 18 to 64 years	Face-to-face	84	200	English	REALM Family	Objective	Words pronounced correctly received a score of 1, and mispronounced or not attempted words received a score of 0.
Stucky et al., [37]	TS-REALD	2011	North Carolina	Pronunciation	Adults	Face-to-face	11	1405	English	REALM Family	Objective	Possible range: 0–9 (raw score). For interpretation, raw scores are transformed
Wong et al., [38]	HKREALD-30	2012	Hong Kong	Comprehension, Numeracy	Adults: 18 to 64 years, Adolescents: 10 to 17 years	Paper and pencil, Face-to-face	52	200	Cantonese	REALM Family	Objective	Possible range: 0–52, ↑scores = ↑ Oral HL
Lee et al., [39]	OHLA-S	2012	North Carolina	Word recognition section and a comprehension	aged 18 or older but less than 80 years	Face-to-face	24	405	Spanish and English	REALM Family	Objective	Sum score (0–24)
Gironda et al., [40]	REALMD-20	2013	USA	Pronunciation	least 18 years of age	Face-to-face	20	200	English	REALM Family	Objective	Sum score (0–20)
Wong et al., [41]	HKOHLAT-P	2013	Hong Kong	Pronunciation	Adults:aged 45–64 years	Face-to-face	30	200 pairs of paediatric dental patients	Hong Kong	TOFHLA Family	Self-reported	Total score range of 0–52, with higher scores indicating better functional OHL.
Jones et al., [42]	HeLD	2013	Australians	Comprehension, Numeracy	Older Adults: 65+ years, Adults: 18 to 64 years, Adolescents: 10 to 17 years	Paper and pencil, Face-to-face	29	209	English		Self-reported	NR
Naghibi Sistani et al., [43]	OHL-AQ	2013	Tehran (Iran)	Reading comprehension,numeracy, literacy and decision making	adults aged between 18 and 65 years	Face-to-face	17	97	Persian	TOFHLA Family	Self-reported	Possible range: 0–17 Inadequate, 0–9; marginal, 10–11; and adequate, 12–17.
Tadakamadla et al., [44]	AREALD-30	2014	Saudi Arabia	Pronunciation	aged over 25 years	Face-to-face	30	177	Arabic	REALM Family	Objective	Sum score (0–30)
Pakpour et al., [45]	IREALD-99	2014	Iran	Pronunciation	Adults: 18 to 64 years	Face-to-face	99	421	Persian	REALM Family	Objective	Sum score (0–99)
Junkes et al., [46]	BREALD-30	2015	Brazilian	Pronunciation	aged 18 to 75 years	Face-to-face	30	258	Brazilian-Portuguese	REALM Family	Objective	Sum score (0–30)
Peker et al., [47]	TREALD-30	2017	Turkish	Pronunciation	Adults: 18 to 64 years	Face-to-face	30	127	Turkish	REALM Family	Objective	Sum score (0–30)
Cruvinel et al., [48]	REALMD-20	2017	Brazilian	Pronunciation	Older Adults: 65+ years, Adults: 18 to 64 years	Face-to-face	20	200	Portuguese	REALM Family	Objective	NR
Bado et al., [49]	OHLA-B	2017	Brazilian	Pronunciation and comprehension	Adults	Paper and pencil, Face-to-face	30	20	Portuguese	REALM Family	Objective	Sum score (0–30)
Cartes-Velásquez and Luengo Machuca, [50]	OHLI-cl	2017	Chilean	Comprehension, Numeracy, General	Adults: 18 to 64 years	Paper and pencil, Face-to-face	57	482	Spanish	–	Objective	Possible range: 0–100 (comprehension score × 1.31, numeracy score × 2.63), with 0–59: inadequate HL, 60–74: marginal HL, and 75–100: adequate HL
Cartes-Velásquez and Luengo-Machucaa, [51]	Span-REALD-30	2018	Chilean	Pronunciation, Numeracy, General, Comprehension	Adults: 18 to 64 years	Face-to-face	30	482	Spanish	–	Objective	NR

Of the 21 tools examined, 16 tools had evaluated the word recognition (short form or quick estimate) [31–35, 37–51], and only one study had examined the “decision-making” dimension [43]. Dimensions of evaluation, responsibility and interaction had not been measured in any instrument (Table 3).

**Table 3:**
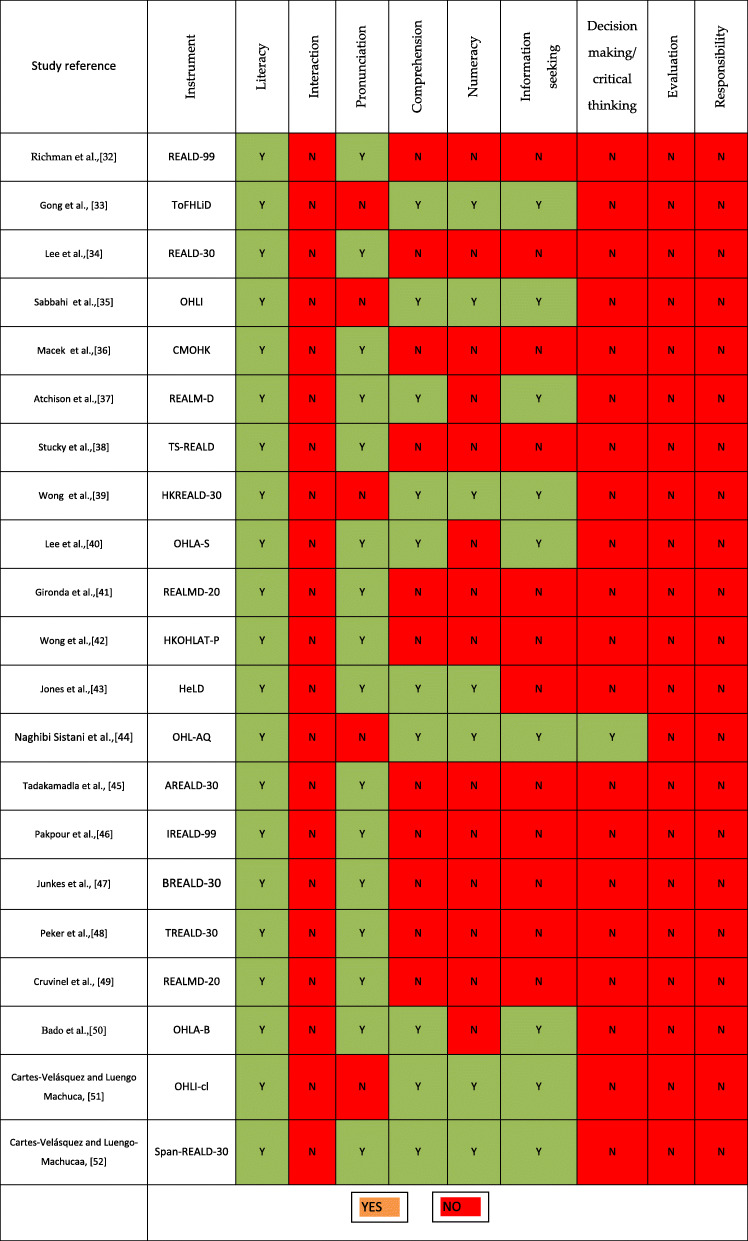
Dimensions assessed in health literacy measures

### Methodological quality of the studies

The results of evaluation of COSMIN checklist are presented in Table 4. Also a summary of the quality of the domains examined on the basis of a checklist COSMIN for oral health assessment tools reported in the Supplementary material 3.

**Table 4:**
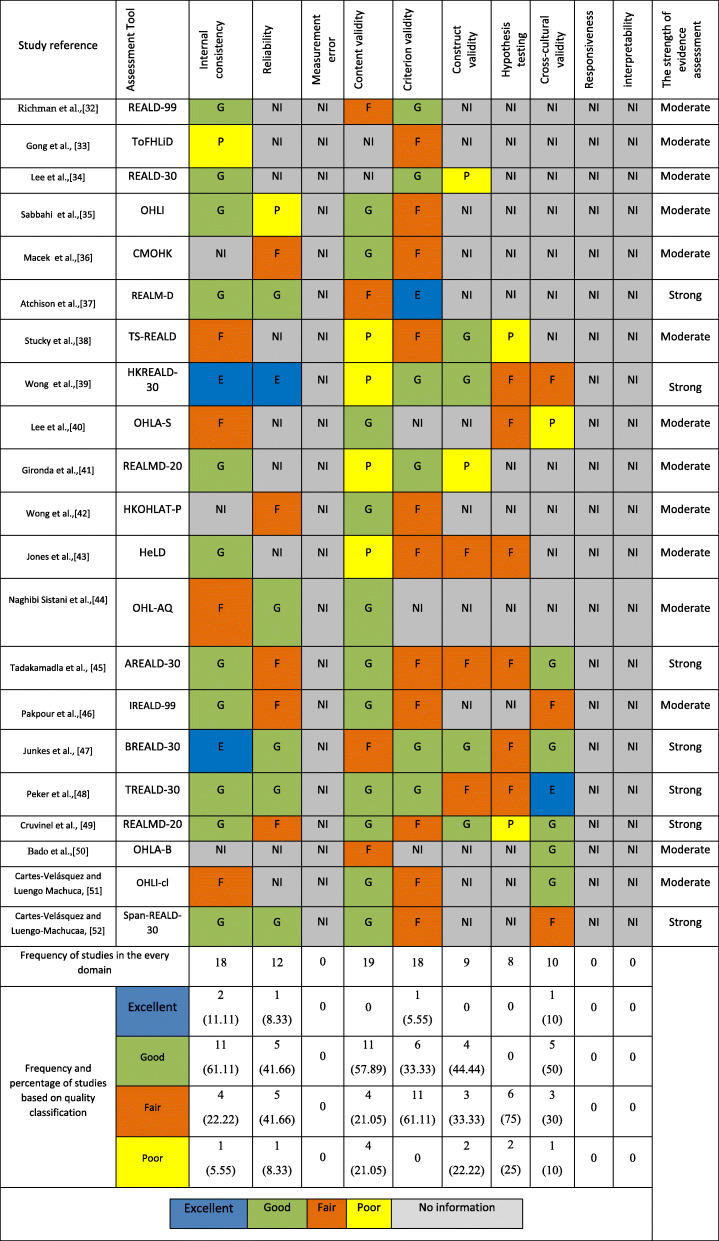
Results of Consensus-based Standards for the selection of health Measurement Instruments (COSMIN) Checklist

### Studies that did not report information were ignored

The results of methodological quality evaluation of the tools showed that, out of the 21 tools examined, 9 tools at least in one dimension were in the category of poor, which indicated the poor quality of that area [32–34, 36–40, 42, 48]. The results of tool review using the COSMIN checklist showed that, 19 studies at least in one dimension had a “fair” quality, which indicated the suspected methodological quality [31, 32, 34–39, 41–51]. Also, 20 and 4 articles at least in one dimension had a “good” [31, 33–51] and “excellent” [36, 38, 46, 47] quality, respectively.

Four tools, by examining seven domains, had paid the most attention to the domains in the psychometric section [38, 44, 46–48], and the two tools of ToFHLiD and OHLA-B had evaluated the minimum domains in the COSMIN checklist [32, 49].

In the area of internal consistency, all tools were evaluated except for three tools [35, 41, 49]. The range of Cronbach alpha score in the reviewed studies varied from 0.63 [32] to 0.91 [47]. The “adequate” criterion for this dimension was Cronbach’s alpha of ≥0.70, which was obtained in other studies except for one study (43). In other studies, the standard range was obtained. Reliability was also investigated in 12 studies [34–36, 38, 41, 43, 44, 46–48, 51]. The most common statistical methods used to evaluate this domain were t-retest and ICC. Construct validity was also evaluated in 9 studies [33, 37, 38, 40, 42, 44, 46–48]. Confirmatory and exploratory factor analysis were the most common statistical methods used to determine structural validity. In this section, the factor analysis with total variance of more than 50% was considered as the adequate criterion. The measurement error, responsiveness, and interpretability domains were not investigated in any tool.

The results of present study showed that, the highest percentage of “excellent” and “good” quality was related to the area of internal consistency, the “fair” quality was related to the area of criterion validity, and the “poor” quality was related to the area of hypothesis testing.

## Discussion

In this study, we attempted to examine tools that measure OHL. Based on the results of present study, the tools were different in terms of what concept of oral and dental health literacy they were measuring. They were also different in terms of items such as scoring, attention to the clinical or health dimension, target group, sample size related to the design and psychometric, and considering the dimensions of oral and dental health literacy.

Based on the results of present study, most oral and dental health literacy tools merely measure the primary skills of OHL including word recognition, reading comprehension, and computation. Based on what Sørensen et al. [23] have considered for a complete HL tool, there is still a considerable shortcoming in these tools in terms of the accurate measuring of oral and dental health literacy, despite many tools that are available in this regard.

According to a conceptualization method by Nutbeam (2000), HL has been defined at three levels [52]. “Basic or functional health literacy” deals with basic reading and writing skills to function effectively in health domain. “Communicative or interactive health literacy” entails more advanced literacy and social skills and enables one to actively participate in healthcare, extract information and infer meaning from different forms of communication and use information to change situations. “Critical health literacy” enables one to critically analyze information and take part in activities that help to overcome structural barriers to health. The last two levels, interactive and critical, specifically address health literacy and health promotion through links to self-efficacy and empowerment. A cross-comparison of the three levels by Nutbeam using the different available instruments in oral/dental health domain showed that the majority of the existing instruments are focused on basic and functional health literacy. To improve oral/dental health literacy, availability of comprehensive instrumentation can fill the gap in the related literature. It needs to be followed by relevant interventions to improve oral/dental health. Thus, concerning the oral/dental health measurement instruments, researchers are suggested to tap on the other levels of health literacy defined by Nutbeam (communicative and critical) to evaluate the content of oral/dental health literacy measurement instruments. In another framework reported by IOM, oral/dental health literacy is affected by different variables the most important of which is education [53]. This variable has been a key correlate of low health literacy. Moreover, the key role of education has been pinpointed in different health promotion declarations from Ottawa [54] to Shanghai [55] by many researchers and stakeholders. Health literacy influenced by education (directly or indirectly) can be achieved in different settings (school, university, workplace, etc.). Therefore, to better evaluate health literacy and consequently the relevant interventions to better health literacy, one factor that requires particular attention is the tailoring of health literacy measurement instruments to different target settings and sub-groups.

Various approaches to literacy tools are among issues that contribute to the inadequacy of HL tools. In other words, the basis for design and development of HL tools (including oral and dental health literacy) is either theoretical or practical, but in practice, this indicator is not measured by a fixed or definite approach or concept. These differences lead to different outcomes and provide scholars and decision makers with a wide range of comparisons and conclusions.

In this study, we also found differences in the methodology, measurement and psychometric of oral and dental health literacy tools. The results showed that, there is no comprehensive tool to examine all dimensions of COSMIN checklist. None of the tools had examined or reported the areas of responsibility, measurement error, and interpretability.

Health measurement tools should consider two areas of validity and reliability to ensure the accuracy of diagnosis and compliance [29]. The results showed that most tools that examine validity and reliability, had a low or fair quality based on the COSMIN methodology. Therefore, considering the importance of HL tools, it is recommended to pay more attention to the psychometric evaluation of the tools. The risk of inappropriate evaluation and misdiagnosis can be affected by the use of a tool without a solid validity and reliability. The most important consequences of using such tools include the increased likelihood of misinterpretation and incorrect reporting of research results. Since oral and dental health literacy is very important both in the field of treatment and prevention, specific attention must be paid to the areas of validity and reliability when designing and developing a tool in order to reduce adverse outcomes, undesirable treatment planning and inappropriate allocation of resources, including the incorrect provision of preventive and restorative interventions. The results of this study can be used to help researchers select a desirable benchmark for their individual research goals. However, it should be noted that the psychometric properties of the tool should be re-implemented for every new setting, sample, or cultural context [56].

### Practice implications

Since oral and dental health literacy tools are still being developed and designed, the relevant stakeholders including health professionals, treatment team and researchers are recommended to evaluate the tools available to synchronize them with the conceptual and scientific perspective related to their specialized goals. For an oral and dental health literacy tool that is tailored to the target group and the subject matter, it is vital to measure the domains of oral and dental health literacy.

In some cases, depending on the purpose of the research, rapid estimation tools can also be useful. In most cases however, functional tools can be more effective as they provide deeper knowledge on oral and dental health literacy of target group. Whenever possible, the use of comprehensive tools (gold standard) that can cover all aspects (including content and psychometric) are useful in acquiring a deep comparative knowledge on the dimensions of oral and dental health literacy or comparison with other tools.

### Study limitations

One of the limitations of this study was that, only studies in English were included in the review. The COSMIN checklist could also be considered as another limitation of this study, as in this checklist, the validity of criteria requires a golden standard, and this is while that, there is currently no standardized tool for measuring oral and dental health literacy, and the existing studies on oral and dental health literacy are used to assess the validity of the criteria. Individual subjectivity can also play an important role in the search, data extraction and synthesis of results, so to prevent the bias, two authors were used to perform the above processes.

## Conclusion

The findings of this study showed that some aspects of oral and dental health literacy are being ignored in the existing tools. On the other hand, some areas of psychometric evaluation of the tools are not being considered, which could jeopardize the credibility of existing tools. Other findings of this study include the deficiencies in the validation methodology of the tools. Therefore, the authors of present study emphasize on the necessity to design and develop a comprehensive tool and take into account two characteristics of simplicity and briefness for international use. Because it is only then that, the tool can be used to transform oral and dental health literacy into a comprehensive and usable index for monitoring the world’s health system (in oral health).

## Supplementary information

**Additional file 1.** PRISMA 2009 Checklist.

**Additional file 2: Supplementary material 2.** Criteria for quality rating of measurement properties.

**Additional file 3: Supplementary material 3.** A summary of the quality of the domains examined on the basis of a checklist COSMIN for oral health assessment tools.

## Data Availability

The datasets used and analyzed during the current study are available from the corresponding author on reasonable request.

## Abbreviations

COSMIN: Consensus based Standards for the selection of health status Measurement Instruments
HL: Health literacy
OHL: Oral health literacy
PRISMA: Preferred Reporting Items for Systematic Reviews and Meta-Analyses
