# Supplementary material for: Systematic review of the tools of oral and dental health literacy: assessment of conceptual dimensions and psychometric properties
Source: BMC Oral Health. 2020 Jul 3;20:186. doi: 10.1186/s12903-020-01170-y (PMC7333397; doi:10.1186/s12903-020-01170-y)
Supplement: Supplementary file 2 — Additional file 2: Supplementary material 2. Criteria for quality rating of measurement properties. [file 12903_2020_1170_MOESM2_ESM.docx]

**Supplementary material 2**: Criteria for quality rating of measurement properties

| **Measurement properties** | **Criteria for ‘adequate’ rating** |
| --- | --- |
| Internal consistency | Cronbach’s alpha is ≥ 0.70. |
| Reliability | Interclass correlation coefficient for continuous variables or weighted kappa for ordinal variables is ≥ 0.70. |
| Measurement error | Smallest detectable change (SDC) is less than minimal important change (MIC) or if the MIC is outside the limit of agreement. |
| Content validity | The items are relevant to the construct being measured. |
| Structural validity | Factor analysis shows that the instrument items explain more than 50% of the total variance. |
| Hypothesis testing | Correlation with other instruments that assess oral health literacy (convergent validity) is ≥ 0.50, or ≥ 75% of the findings are accordance with the a priori hypothesis. If the hypothesis tested for a correlation with another construct other than population target (divergent validity), a score of ‘unclear’ is given. |
| Cross-cultural validity | Factor analysis, logistic regression, or item response theory techniques detected differential item function between two or more language groups for the instrument. |
| Responsiveness | Correlation with the change score of an instrument assessing oral health literacy is ≥ 0.5, or if ≥ 75% of the findings were in accordance with a priori defined hypotheses, or if the area under the receiver operator characteristic curve (ROC) ≥ 0.70. |
| Interpretability | information on floor or ceiling effects and the minimal important differences are provided. |
