# Supplementary material for: Systematic review of the tools of oral and dental health literacy: assessment of conceptual dimensions and psychometric properties
Source: BMC Oral Health. 2020 Jul 3;20:186. doi: 10.1186/s12903-020-01170-y (PMC7333397; doi:10.1186/s12903-020-01170-y)
Supplement: Supplementary file 3 — Additional file 3: Supplementary material 3. A summary of the quality of the domains examined on the basis of a checklist COSMIN for oral health assessment tools. [file 12903_2020_1170_MOESM3_ESM.docx]

**Supplementary material 3. A summary of the quality of the domains examined on the basis of a checklist COSMIN for oral health assessment tools.**

| **Author** | **Instrument** | **Content validity** |
| --- | --- | --- |
|  |  |  |
| Richman et al(2007) | REALD-99 | All words were taken from the American Dental Association's Glossary of Common Dental Terminology. Words or terms were also included from patient brochures and written materials provided at the University of North Carolina at Chapel Hill (UNC-CH) School of Dentistry |
| Lee et al(2007) | REALD-30 | This investigation is based on an item response theory analysis. |
| Gong et al.(2007) | ToFHLiD | A panel of pediatric and public health dentists reviewed patient education and instructional materials used in the UNC-Chapel Hill pediatric dental clinic and selected a sample from these materials to use in the TOFHLiD. |
| Sabbahi et al(2009) | OHLI | For the generation of items, different dental patient educational materials and text types were reviewed. These included pamphlets, brochures and on-line materials, preoperative and postoperative instructions for different dental procedures, labels and instructions for commonly prescribed drugs in dentistry, and patient registration and appointment forms. The materials and texts, and the activities associated with them, were drawn from various dental health related contexts and had reading levels similar to materials used for the Test of Functional Health Literacy of Adults (TOFHLA), on which the Oral Health Literacy Instrument (OHLI) was modelled. |
| Macek et al(2010) | CMOHK | As a final check of face and content validity, a group of 15 practicing dentists in Maryland were asked to comment on the multiple-choice items. Their input was used for final editing. No additional pilot-testing was conducted. |
| Wong et al.(2012) | HKREALD-30 | A panel consisting of pediatric and public health dentists reviewed patient education and instructional materials used in the UNC-CH pediatric dental clinic and selected a sample from these materials for use in TOFHLiD. |
| Lee et al.(2012) | OHLA-S | An expert panel was formed to develop the OHLA instrument through a Delphi process. The panel consisted of nine members (six females and three males): three bilingual native Spanish-speaking dentists, three bilingual laypeople; and three who were not bilingual, but had extensive experience working with Spanish speakers in educational dental settings, and included two dentists and one sociologist who was also an expert in health literacy. To reflect our target population, the bilingual dentists and laypeople on the panel were of Mexican and Central American descent or had extensive experience working with these populations. |
| Gironda et al.(2013) | REALMD-20 | Unknown |
| Wong et al.  Bridges et al  (2013) | HKOHLAT-P | for the generation of items and to develop the HKOHLAT-P, the materials were collected from different dentally related areas, which includes oral health TV and radio programs from Hong Kong local broadcasts, oral health education videos and oral health instructions in Prince Philip Dental Hospital (Faculty of Dentistry, The University of Hong Kong), oral health brochures from the Department of Health (Oral Health Education Unit) in Hong Kong Special Administrative Region, and Hong Kong local newspapers. These materials were then used to develop a Chinese corpus database in dentistry. The materials and texts and the activities associated with them were drawn from various dental health-related contexts and were selected to have reading levels similar to materials used for TOFHLiD and OHLI, from which we modelled some of the items for the HKOHLAT-P. Original items were developed to expand the text types of reading stimuli and give variety in item design. |
| Jones et al.(2013) | HeLD | The HeLD was modelled after the Health Literacy Measurement Scale (HeLMS). Theoretical constructs and items of the HeLMS were adapted for use in an oral health context with the guidance of oral health experts. |
| Stucky et al.(2011) | TS-REALD | This investigation is based on an item response theory analysis of the REALD-30. |
| Junkes et al(2015) | BREALD-20 | Based on standard recommendations, the translation and cross-cultural adaptation of the REALD-30 were initially performed by two independent translators (a Brazilian fluent in English and a native English speaker fluent in Portuguese) with experience in the translation of health questionnaires. |
| Parker et al.(2017) | TREALD-30 | Two native Turkish speakers translated the original English REALD-30 into Turkish. The two versions were then synthesized and back translated to ensure cultural equivalence. Next, an expert committee of biostatisticians, oral health professionals, linguists, and translators developed a "pre-final" version, which was then pilot-tested among a convenience sample of 35 adult dental patients. |
| Tadakamadla et al.(2014) | AREALD-30 | An expert panel was convened with three bilingual individuals (two of them were dental professionals) to resolve the discrepancies between the independently translated versions. In addition, an independent professional translator back-translated the Arabic version into English and no discrepancies existed between the original and back-translated English versions of AREALD-99 and A-OHIP-14. AREALD-99 and A-OHIP-14 questionnaire were pilot tested on a convenience sample of twenty patients visiting the dental clinics to assess face and content validity. |
| Cruvinel et al.(2017) | REALMD-20 | The original REALMD-20 was translated into Brazilian Portuguese. A few terms were replaced with culturally appropriate, equivalent terms prior to a pre-test of the tool conducted through face to face interviews. Volunteers' feedback was used to evaluate the difficulty of each item. Items were then arranged in ascending order of difficulty. Finally, the tool was back-translated to ensure consistency with the original version. |
| Bado et al.(2017) | OHLA-B | Initial translation into Portuguese language by two Spanish teachers, back translation into Spanish by two native Spanish speaking, review by the committee, and pre-test. |
| Sistani et al.(2013) | OHL-AQ | Eight experts (six oral public health specialists, a methodologist, and a health education expert) appraised the questionnaire by scoring relevancy, clarity, simplicity, and necessity of the items in order to calculate the content validity index (CVI) and content validity ratio (CVR). |
| Cartes-Velasquezet al. | OHLI-cl | The development of the tool involved two independent translation and back translation of the original OHLI from English to Spanish and vice-versa by two native Spanish language professionals proficient in English. The two independent versions of translation where then revised by a third bilingual professional to achieve a consensus. The final version of the tool was revised by a panel of experts in dental public health. |
| Cartes-Velasquez et al. | Span-REALD-30 | The development of the tool Spanish REALD-30 included translation from English to Spanish, there was a pilot study and, and the final version was revised by experts. |
| Pakpour et al. | IREALD-99 | The IREALD-99 was translated into the Persian language, then back translated by native English speakers. The Persian version was then pilot tested among 12 adults to identify any problems with the translation. |
| Atchison et al. | REALM-D | Used the REALM as a starting point and added additional dental/medical terminology per a convenience sample of dental students and patients. |

| **Author** | **Instrument** | **Criterion validity** | |
| --- | --- | --- | --- |
|  |  | **Concurrent criterion validity** | **Predictive criterion validity** |
| Richman et al(2007) | REALD-99 | REALD-99 was correlated positively with the REALM (Pearson correlation coefficient = 0.80, P< 0.05). REALD-99 was associated with parents' Oral Health Impact Profile Short Form score (OHIP-14) score in multi-variate analysis. | REALD-99 was correlated with oral health quality of life, but not self-perceived oral health status in multivariate analysis, providing mixed results. |
| Lee et al(2007) | REALD-30 | REALD-30 was significantly (p= 0.05) positively correlated with the REALM (0.86) and TOFHLA (0.64). | REALD-30 associated with oral health-related quality of life, but not with perceived dental health status |
| Gong et al.(2007) | ToFHLiD | Unknown | TOFHLiD scores were associated with the child's oral health status and the caregivers' OHIP-14 scores (P, 0.05) in bi-variate analyses, but not the oral health status of the caregiver. |
| Sabbahi et al(2009) | OHLI | Convergent: TOFHLA r=0.61, and Discriminate: Oral Knowledge r=0.57 | Unknown |
| Macek et al(2010) | CMOHK | Chi-square/Fisher’s exact test analysis confirmed that the CMOHK and REALM were significantly associated with one another (Fisher’s exact test P-value < 0.01) whereas the CMOHK and Short TOFHLA were not (chi-square P-value = 0.62). | Unknown |
| Wong et al.(2012) | HKREALD-30 | There was a significant correlation (P < 0.01) between reading habits and HKREALD-30. Indicating that the more hours the participants spent in reading (Spearman’s rho = 0.389 for print materials and 0.278 for digital materials), the higher the HKREALD-30 scores they obtained. | The predictive validity of REALD-30 was partially supported by a positive association (P < 0.05) with the short form Oral Health Impact Profile (OHIP- but the correlation with self-perceived dental health status was not statistically significant. |
| Lee et al.(2012) | OHLA-S | Unknown | Unknown |
| Gironda et al.(2013) | REALMD-20 | Unknown | The REALMD-20 was highly correlated with coefficients ranging from rs = 0.90 to rs = 0.93.Correlations between instruments and two single-item indicators were weakly, but significantly correlated with positive correlations between rs = 0.25 and rs = 0.32 (P < 0.001) for confident filling out medical forms, and negative correlations between rs =-0.25 and rs =-0.34 (P < 0.001) for needing help reading hospital material. |
| Wong et al.  Bridges et al  (2013) | HKOHLAT-P | Concurrent: Self-Reading r=0.43 and Reading to child r=0.12 (N/S); Convergent: TOFHLiD r=0.39 and HKREALD-30 r=0.36 | Predictive: ECOHIS, r=-0.24 |
| Jones et al.(2013) | HeLD | Unknown | HeLD scores were significantly correlated with toothbrush ownership (r=0.14, p<0.01). |
| Stucky et al.(2011) | TS-REALD | Convergent: REALD-30 r=0.96 and NVS r=0.51; Concurrent: OHIP-14§, β= -0.74 and SRDHS¶, β=0.10 | Unknown |
| Junkes et al(2015) | BREALD-20 | Unknown | Regarding predictive validity, no correlation was found between the BREALD-30 and OHIP-14 scores (rs = -0.080; p = 0.198), but the BREALD-30 score was statistically associated with self-rated oral health and respondent’s assessment of his/her child’s oral health in the bivariate analysis (Mann Whitney, p = 0.003 for both variables). |
| Parker et al.(2017) | TREALD-30 | Positive correlation between TREALD-30 and REALM (r=0.73, p<0.01) as well as two single item literacy screeners (r=0.69, p<0.01 for reading ability of hospital materials; r=0.59, p<0.01 for perceived confidence in completing medical forms). | Positive correlation between TREALD-30 and education (r=0.78, p<0.01). |
| Tadakamadla et al.(2014) | AREALD-30 | AREALD-30 correlated significantly and positively with AREALD-99 (0.959, P<0.01), A-OHIP-14 99 (- 0.105, P<0.01), self-perceived oral health status (0.136, P<0.01), and dental visiting habits (-0.142, P<0.01). | Unknown |
| Cruvinel et al.(2017) | REALMD-20 | The Portuguese REALMD-20 was positively correlated with BREALD-30 (Rs=0.73, p>0.001) and BNFLI (Rs=0.60, p<0.001). | Portuguese REALMD-20 scores were significantly higher among health professionals and individuals of higher educational attainment. |
| Bado et al.(2017) | OHLA-B | Unknown | Unknown |
| Sistani et al.(2013) | OHL-AQ | Unknown | Unknown |
| Cartes-Velasquezet al. | OHLI-cl | Unknown | To determine the predictive validity, the OHLI scores were correlated with clinical variables (DMFT, CPI and OHIS) and OHRQoL (OHIP-49sp). All correlations were statistically significant (P < 0.01) and showed weak-to-moderate predictive validity. |
| Cartes-Velasquez et al. | Span-REALD-30 | Unknown | Pearson´s and Spearman´s correlations were <= -0.138 for predictive validity. |
| Pakpour et al. | IREALD-99 | IREALD-99 scores positively correlated to Test of Functional Health Literacy in Dentistry (TOFHLiD) scores (rh=0.72, p<0.01). | Unknown |
| Atchison et al. | REALM-D | Assessed in terms of correlation with the 66-item REALM (r=0.99) and single-item indicators of health literacy:1) how often the participant needed help reading medical forms; 2) how confident the patient was in filling out medical forms, and patient sociodemographic. | Among the variables that were associated with health literacy at a bivariate level, race, education and English as a main language remain predictive of health literacy. A trend (P=0.063) is noted for reporting confidence in filling out medical forms. To further assess the association between health literacy and confidence in filling out forms, a separate regression analysis predicting confidence using race, gender, and education level as covariates and health literacy as a predictor was conducted. Health literacy was found to be the only significant predictor (b=0.50, standard error = 0.21, P=0.02) of confidence filling out medical forms. |

| **Author** | **Instrument** | **Convergent validity** |
| --- | --- | --- |
|  |  |  |
| Richman et al(2007) | REALD-99 | Unknown |
| Lee et al(2007) | REALD-30 | Unknown |
| Gong et al.(2007) | ToFHLiD | Unknown |
| Sabbahi et al(2009) | OHLI | Unknown |
| Macek et al(2010) | CMOHK | Unknown |
| Wong et al.(2012) | HKREALD-30 | It was found that HKREALD-30 and TOFHLiD were highly correlated (Spearman’s rho = 0.693, P < 0.01). |
| Lee et al.(2012) | OHLA-S | Consistently statistically observed significant associations between the OHLA-S and the variable used to determine convergent validity (TOFHLA). |
| Gironda et al.(2013) | REALMD-20 | Unknown |
| Wong et al.  Bridges et al  (2013) | HKOHLAT-P | Unknown |
| Jones et al.(2013) | HeLD | Unknown |
| Stucky et al.(2011) | TS-REALD | Unknown |
| Junkes et al(2015) | BREALD-20 | The BREALD-30 demonstrated satisfactory convergent validity, as the scores correlated with the level of general literacy measured by the NFLI (rs = 0.593; p < 0.001) and educational attainment (rs = 0.541; p < 0.001). |
| Parker et al.(2017) | TREALD-30 | Unknown |
| Tadakamadla et al.(2014) | AREALD-30 | Unknown |
| Cruvinel et al.(2017) | REALMD-20 | Unknown |
| Bado et al.(2017) | OHLA-B | Unknown |
| Sistani et al.(2013) | OHL-AQ | Unknown |
| Cartes-Velasquezet al. | OHLI-cl | Unknown |
| Cartes-Velasquez et al. | Span-REALD-30 | Unknown |
| Pakpour et al. | IREALD-99 | Unknown |
| Atchison et al. | REALM-D | Unknown |

.....................................................

| **Author** | **Instrument** | **Reliability notes** |
| --- | --- | --- |
|  |  |  |
| Richman et al(2007) | REALD-99 | Cronbach's alpha: 0.86 |
| Lee et al(2007) | REALD-30 | Cronbach's alpha: 0.87 |
| Gong et al.(2007) | ToFHLiD | Cronbach's alpha: 0.63 |
| Sabbahi et al(2009) | OHLI | Cronbach’s alpha was 0.89  The ICC values for the total OHLI, reading comprehension and numeracy sections (>0.6) |
| Macek et al(2010) | CMOHK | Cronbach alpha = 0.74 |
| Wong et al.(2012) | HKREALD-30 | The ICC value of HKREALD-30 was 0.78 (confidence intervals = 0.53–0.91), which indicated test–retest reliability, was good (ICC = 0.61–0.80).22 The internal consistency of HKREALD-30 was acceptable (Cronbach’s alpha > 0.7) because the value of Cronbach’s alpha was 0.84. |
| Lee et al.(2012) | OHLA-S | Cronbach Alpha of 0.70-0.80 |
| Gironda et al.(2013) | REALMD-20 | Cronbach Alpha : 0.86 |
| Wong et al.  Bridges et al  (2013) | HKOHLAT-P | Test-retest correlation: Test-retest: ICC=0.63 |
| Jones et al.(2013) | HeLD | Cronbach's alpha: 0.91 |
| Stucky et al.(2011) | TS-REALD | IRT: aΔ=2.91 to 1.09, with mean =2.12 and SD=0.44; b□ =1.97 to -2.93, with mean =0-.02 and SD=1.42 |
| Junkes et al(2015) | BREALD-20 | Cronbach's alpha ranged from 0.88 to 0.89 when words were deleted individually. The analysis of test-retest reliability demonstrated excellent reproducibility [ICC = 0.983 (95% CI: 0.963 to 0.993) and Kappa coefficients ranging from moderate to nearly perfect (0.42–1.00)] |
| Parker et al.(2017) | TREALD-30 | Test-retest correlation: 0.99 , Cronbach's alpha: 0.91 |
| Tadakamadla et al.(2014) | AREALD-30 | The internal consistency of both the Arabic word recognition instruments was good, Cronbach’s alpha was found to be 0.89 and 0.91 for AREALD-30 and AREALD-99 respectively. The ICC used to examine the test-retest reliability was higher than 0.90 for all the instruments, indicating that there was an excellent agreement between the repeated administrations. |
| Cruvinel et al.(2017) | REALMD-20 | Reliability: Test-retest correlation: ICC = 0.73; 95% CI, Cronbach's alpha: 0.789 |
| Bado et al.(2017) | OHLA-B | Unknown |
| Sistani et al.(2013) | OHL-AQ | The Cronbach’s alpha coefficient was found to be 0.72, and the test–retest analysis demonstrated an ICC value of 0.84, both indicating satisfactory results. |
| Cartes-Velasquezet al. | OHLI-cl | Cronbach's alpha: 0.887 |
| Cartes-Velasquez et al. | Span-REALD-30 | Cronbach's alpha: 0.876, Intra-class correlation coefficient was 0.789 for reliability |
| Pakpour et al. | IREALD-99 | Test-retest correlation: 0.97, Cronbach's alpha: 0.98 |
| Atchison et al. | REALM-D | Test-retest correlation: high correlation found between initial and follow-up total scores (r= 0.95) as well as between each list (L1=0.93, L2=0.95,L3=0.87)  Cronbach's alpha: 0.958 |

| **Author** | **Instrument** | **Construct validity** |
| --- | --- | --- |
|  |  |  |
| Richman et al(2007) | REALD-99 | Unknown |
| Lee et al(2007) | REALD-30 | The results of the factor analysis of the words in REALD-30 showed a clear dominance of a single factor and the presence of a second. The eigenvalue for the first factor (8.78) was more than four times larger than that of the second (2.10), which was four times that of the third factor (<0.5). |
| Gong et al.(2007) | ToFHLiD | Unknown |
| Sabbahi et al(2009) | OHLI | Unknown |
| Macek et al(2010) | CMOHK | Unknown |
| Wong et al.(2012) | HKREALD-30 | The results of factor analysis, 47 items of the HKREALD-99 had factor loadings that did not exceed 0.3 for the rotated factor. Therefore, items such as ‘‘splint’’ and ‘‘prescription’’ were eliminated from the scale. For the 52 items remaining in the scale, 17 were deleted according to Rasch analysis because their infit/outfit ZSTD was not within the range of) 2 and 2, or the infit/outfit MNSQ was outside the range of 0.70 and 1.30. In this procedure ‘‘habits’’ (outfit MNSQ = 0.41) and ‘‘analgesia’’ (outfit ZSTD = 2.38, outfit MNSQ = 2.78) were two examples of deleted items. The remaining 35 items were further examined by their frequency counts in the Chinese corpus database of dentistry developed in this study. The frequency counts of ‘‘copayment’’, ‘‘coverage’’, ‘‘biopsy’’, ‘‘malignant’’, and ‘‘neuralgia’’ were less than 0.01% and therefore eliminated. |
| Lee et al.(2012) | OHLA-S | Unknown |
| Gironda et al.(2013) | REALMD-20 | The second step was to re-run the factor analysis using a varimax rotation method, with the remaining 11 dental terms (anesthetic, abscess, amalgam, caries, calculus, dentures, gingivitis, extraction, insurance, hygiene, and periodontitis) and adding the 8REALM-Rmedical terms (fatigue, directed, jaundice, allergic, constipation, anemia, colitis, osteoporosis), plus depression. Two factors emerged, explaining 45 percent of the total variance of the 20-item list with 7.14 and 1.77 eigenvalues (see Table 1). The first factor consists of 10 less difficult items (abscess, dentures, hygiene, insurance, extraction, fatigue, directed, allergic, constipation, and depression) with 5 dental terms and 5 medical/mental health terms. |
| Wong et al.  Bridges et al  (2013) | HKOHLAT-P | Unknown |
| Jones et al.(2013) | HeLD | Exploratory factor analysis extracted seven inter-correlated factors with eigenvalues >1, Kaiser-Meyer-Olkin´s test: 0.839 , Bartlett´s test of sphericity: <0.011 |
| Stucky et al.(2011) | TS-REALD | After removing the three items, a 27-item CFA model was fit to the data (including a residual correlation between the item pair brush and floss). The resulting model was found to closely fit the data according to commonly used assessments of model fit (16–18): v2 ð114Þ = 613, CFI = 0.95, TLI =0.97 RMSEA = 0.056. To ensure unidimensionality, the item brush was set aside from the single item pair, resulting in 26 unidimensional items to be calibrated. |
| Junkes et al(2015) | BREALD-20 | Regarding construct validity, the exploratory factor analysis of the words on the BREALD30 demonstrated the undeniable predominance of one factor. The eigenvalue for the first factor (7.36) was approximately fourfold greater than that for the second factor (1.88), which was similar to the eigenvalue for the third factor (1.62). The scree plot also demonstrated the clear predominance of one factor. Based on the Kaiser criterion, only factors with eigenvalues greater than one were extracted; as those with lower values contribute little to explaining the variance in the original variables [22]. Factor I (nine words) accounted for 24.5% of the variance and included the words analgesia, endodontics, malocclusion, abscess, fistula, hyperemia, orthodontics, hypoplasia and apicectomy. Factor II accounted for 6.3% of the variance and included the words teeth, erosion, restoration, biopsy, bruxism, periodontal and film. A minimum of seven factors were necessary to explain 50% of the total variance. |
| Parker et al.(2017) | TREALD-30 | The results of CFA indicated that the two-factor model demonstrated a better fit than did the one-factor model (v2/df¼1.34, CFI¼0.89, IFI ¼0.90, TLI¼0.89, and RMSEA¼0.052). |
| Tadakamadla et al.(2014) | AREALD-30 | CFA showed presence of two factors and in fit mean-square statistics for AREALD-30 were all within the desired range of 0.50 - 2.0 in Rasch analysis. |
| Cruvinel et al.(2017) | REALMD-20 | In the factor analysis, it was observed an adequate sample size (KMO = 0.73) with a non-identity correlation matrix (Bartlett’s test of sphericity, P < 0.001), and no influence of multicollinearity (determinant = 0.004). Six factors with eigenvalues >1.0 were extracted: the factor I (eigenvalue = 4.53) comprised four terms—‘‘Jaundice’’, ‘‘Amalgam’’, ‘‘Periodontitis’’ and ‘‘Abscess’’—accounted for 25.18% of total variance, while the factor II (eigenvalue = 1.88) comprised other four terms—‘‘Gingivitis’’, ‘‘Instruction’’, ‘‘Osteoporosis’’ and ‘‘Constipation’’—accounted for 10.46% of total variance. The first four factors accounted for 52.1% of total variance. |
| Bado et al.(2017) | OHLA-B | Unknown |
| Sistani et al.(2013) | OHL-AQ | Unknown |
| Cartes-Velasquezet al. | OHLI-cl | Unknown |
| Cartes-Velasquez et al. | Span-REALD-30 | Unknown |
| Pakpour et al. | IREALD-99 | Unknown |
| Atchison et al. | REALM-D | Unknown |

| **Author** | **Instrument** | **Cross-cultural validity** |
| --- | --- | --- |
|  |  |  |
| Richman et al(2007) | REALD-99 | Unknown |
| Lee et al(2007) | REALD-30 | Unknown |
| Gong et al.(2007) | ToFHLiD | Unknown |
| Sabbahi et al(2009) | OHLI | Unknown |
| Macek et al(2010) | CMOHK | Unknown |
| Wong et al.(2012) | HKREALD-30 | Three additional sets of self-completed questionnaires were administered to the participants to assess the validity of the translated and modified instrument: (a) the Test of Functional Health Literacy in Dentistry (TOFHLiD);18 (b) a self-report questionnaire developed to collect data on the respondents’ reading habits (namely, the hours spent in personal reading in the past week) involving print (newspapers, books and letters) and digital/screen-based texts (webpages and text messages); and (c) a self-report questionnaire developed to collect the respondents’ background information, such as age, gender, education levels, working status, family incomes and pattern of dental visits. |
| Lee et al.(2012) | OHLA-S | Unknown |
| Gironda et al.(2013) | REALMD-20 | Unknown |
| Wong et al.  Bridges et al  (2013) | HKOHLAT-P | Unknown |
| Jones et al.(2013) | HeLD | Unknown |
| Stucky et al.(2011) | TS-REALD | Unknown |
| Junkes et al(2015) | BREALD-20 | For the determination of conceptual equivalence, a committee of experts in oral health and health education assessed the relevance of the items on the Brazilian-Portuguese language version in comparison to the original English language version. The committee evaluated whether the areas covered by the original instrument regarding the concepts of interest would be relevant and pertinent to the cultural context to which the REALD-30 was being adapted. |
| Parker et al.(2017) | TREALD-30 | Based on standard recommendations, the process of cross-cultural adaptation involves five steps, as follows:  Stage 1 – initial translation. Stage 2 – synthesis of these translations. Stage 3 – Back-translation. Stage 4 – expert committee review  Stage 5 – pretesting |
| Tadakamadla et al.(2014) | AREALD-30 | A pool of “dentistry related words” was constructed by translating English REALD-99 words into Arabic. Along with the REALD-99, 14 item Oral Health Impact Profile (OHIP-14) [17] was translated and the Arabic version of REALD-99 (AREALD-99), AREALD-30 and A-OHIP-14 were obtained. Two bilingual dental professionals with Arabic as their native language independently translated English REALD-99 words and OHIP-14 into Arabic. Translators were instructed to aim at the conceptual equivalence of the words but not the literal translation. An expert panel was convened with three bilingual individuals (two of them were dental professionals) to resolve the discrepancies between the independently translated versions. In addition, an independent professional translator back-translated the Arabic version into English and no discrepancies existed between the original and back-translated English versions of AREALD-99 and A-OHIP-14. AREALD-99 and A-OHIP-14 questionnaire were pilot tested on a convenience sample of twenty patients visiting the dental clinics to assess face and content validity. The participants were queried about the difficulties in understanding the items and any changes required were done accordingly. |
| Cruvinel et al.(2017) | REALMD-20 | The cross-cultural adaptation was performed through conceptual equivalence, verbatim translation, semantic, item and operational equivalence, and back translation. |
| Bado et al.(2017) | OHLA-B | The steps were: initial translation into Portuguese language by two Spanish teachers, back translation into Spanish by two native Spanish speaking, review by the committee, and pre-test. For the pre-test of cross-cultural adaptation, the alternative "did not understand" was added to each item of the tool. The instrument was applied to a sample of 20 adults. Results: In the initial translation, some differences were observed between the translated versions, and after the committee had reviewed these versions, a few words were replaced by other synonyms to enable better understanding of the instrument by the population. When the back-translation was compared with the original version, the results were very satisfactory and there was no need to make any further change or replacement. In the pre-test, the version of the tool Oral Health Literacy Assessment-Brazilian (OHLA-B) was very well understood by the studied population and there was no need for other cultural adaptations. |
| Sistani et al.(2013) | OHL-AQ | Unknown |
| Cartes-Velasquezet al. | OHLI-cl | The process of translation and adaptation was performed according to Beaton et al.21 and Sousa and Rojjanasrirat22. Two native Spanish language professionals proficient in English translated the English version of the OHLI independently. The versions were named TL1 (translation carried out by a dentist) and TL2 (translation carried out by a professional translator without a dental background). Later, a consensus between TL1 and TL2, guided by a third bilingual professional (a dentist), was achieved. The translated version was back-translated by the same method in reverse, generating BT1, BT2 and BT3, respectively. Subsequently, four experts in dental public health reviewed the process for obtaining a preliminary version of the Chilean version of OHLI (OHLI-cl). The whole process was recorded in ad hoc proceedings that were sent, for feedback, to the team who developed the OHLI. |
| Cartes-Velasquez et al. | Span-REALD-30 | Two native Spanish-language professionals proficient in English (a dentist and a professional translator) translated the REALD-30 independently. Later, a consensus guided by a third bilingual professional (dentist) was performed. A final evaluation by four experts in dental public health was made. |
| Pakpour et al. | IREALD-99 | The aim of the translation procedure was to create a translated version in the Persian language that is both conceptually equivalent to the original and easily understood by Iranian people. A forward–backward approach was performed to translate and adapt the IREALD-99 into the Persian language. For the first step, two translators independently translated the questionnaire into Persian. Afterwards, the project manager (AP) compared the translations and reconciled discrepancies. Two translators, who were native English speakers and were fluent in the Persian language, independently translated the interim Persian version into English. The translators were not aware of the English original version. The project manager (AP) compared the English translations with the original questionnaire and reconciled discrepancies. The Persian language was piloted on 12 adults to identify and solve any potential problems in translation (e.g. wording). |
| Atchison et al. | REALM-D | Unknown |
